# Efficacy and safety of fluconazole prophylaxis in extremely low birth weight infants: multicenter pre-post cohort study 

**DOI:** 10.1186/s12887-016-0605-y

**Published:** 2016-05-16

**Authors:** Juyoung Lee, Han-Suk Kim, Seung Han Shin, Chang Won Choi, Ee-Kyung Kim, Eun Hwa Choi, Beyong Il Kim, Jung-Hwan Choi

**Affiliations:** Department of Pediatrics, Inha University School of Medicine, 100 Inharo, Incheon, Nam-gu 22212 Korea; Department of Pediatrics, Seoul National University Children`s Hospital, 101 Daehak-ro, Seoul, Jongno-gu 03080 Korea; Department of Pediatrics, Seoul National University College of Medicine, 103 Daehak-ro, Seoul, Jongno-gu 03080 Korea; Department of Pediatrics, Seoul National University Bundang Hospital, 82, Gumi-ro 173, Bundang-gu, Seongnam, Gyeonggi 13620 Korea

**Keywords:** Fluconazole, Prophylaxis, Invasive candidiasis, Resistance

## Abstract

**Background:**

There have been many studies supporting fluconazole prophylaxis in preterm infants for prevention of invasive fungal infections (IFIs). However, the routine use of fluconazole prophylaxis in neonatal intensive care units (NICUs) raises concerns with respect to resistance development, including the selection of resistant *Candida* species. We aimed to evaluate the efficacy and safety of fluconazole prophylaxis in extremely low birth weight (ELBW) infants.

**Methods:**

An interventional pre-post cohort study at two tertiary NICUs was conducted. Data from two 5-year periods with and without fluconazole prophylaxis (Mar 2008–Feb 2013 and Mar 2003–Feb 2008) was compared. Prophylactic fluconazole was administered starting on the 3rd day at a dose of 3 mg/kg twice a week for 4 weeks during the prophylaxis period.

**Results:**

The fluconazole prophylaxis group consisted of 264 infants, and the non-prophylaxis group consisted of 159 infants. IFI occurred in a total of 19 neonates (4.7 %) during the 10-year study period. Fluconazole prophylaxis lower the fungal colonization rate significantly (59.1 % vs. 33.9 %, *P* <0.001). However, the incidence of IFIs in ELBW infants was not reduced after fluconazole prophylaxis (4.4 % vs. 5.5 %, *P* = 0.80). Rather, although the increase did not reach statistical significance, fluconazole prophylaxis tended to increase the incidence of invasive infections involving fluconazole-resistant *C. parapsilosis* (0 % vs. 41.7 %, *P* = 0.11).

**Conclusions:**

Fluconazole prophylaxis was not efficacious in decreasing IFIs in ELBW infants. There is a need for targeting prophylaxis to greatest risk population and prospective studies to measure the long-term effect of fluconazole prophylaxis on the emergence of organisms with antifungal resistance.

**Electronic supplementary material:**

The online version of this article (doi:10.1186/s12887-016-0605-y) contains supplementary material, which is available to authorized users.

## Background

Preterm infants managed in a neonatal intensive care unit (NICU) are at significant risk of invasive fungal infection (IFI) because of invasive vascular procedures, broad-spectrum antibiotic treatments, prolonged parenteral nutrition, and most importantly, their immature immune systems. For the highest-risk group, extremely low birth weight (ELBW, <1,000 g at birth) infants, IFI is attributable to increase mortality and neurodevelopmental impairment, despite antifungal therapy [1–3]. Several well-designed randomized trials and meta-analyses have shown that antifungal prophylaxis with fluconazole reduces the incidence of IFIs [4–7]. Of note, studies examining fluconazole prophylaxis have been conducted in NICUs with relatively high incidences of IFIs (13–20 %) [4–6]. However, several large cohort studies have reported IFI incidences as low as < 5 % for ELBW infants [3, 8–10]. and a recent study has demonstrated a substantial decrease in IFI incidence in the last 14 years due to improved perinatal and intensive care for preterm infants [11].

Meanwhile, with respect to fluconazole prophylaxis, there has been concern regarding the emergence of resistance to azoles, including the selection for and replacement of resistant *Candida* spp. in preterm infants [12, 13]. Other concerns are short-term drug toxicities and long-term neurodevelopmental consequences of fluconazole when it is used in a “developing immature organism” [14, 15].

We have implemented fluconazole prophylaxis as part of our routine management of ELBW infants since March 2008. We reviewed the clinical data and details of IFIs of infants receiving fluconazole prophylaxis after adopting this practice and compared them to a historical control group of the pre-prophylaxis period. We aimed to evaluate whether fluconazole prophylaxis did decrease the incidence of IFIs in ELBW infants. Moreover, we investigated IFIs involving fluconazole-resistant strains, potential adverse effects of fluconazole, and long-term morbidities as aspects related to drug safety.

## Methods

### Study population and study setting

This interventional pre-post cohort study with a historical control was conducted at two tertiary NICUs, at which the same intervention protocol was applied. The study cohort included all ELBW infants who were born in and admitted to the NICUs of Seoul National University Children’s Hospital and Seoul National University Bundang Hospital between March 2003 and February 2013. We excluded infants prenatally exposed to antifungal agents, those receiving therapeutic antifungal agents within 3 days after birth, and those who died before 3 days of life. The study was approved by the Institutional Review Board of Seoul National University Hospital and informed consent was waived.

As a routine practice protocol, all ELBW infants underwent weekly surveillance culture studies. All clinical and microbiological records for the infants, including surveillance fungal isolate data during the NICU stay, were collected. All of the admitted ELBW infants received fluconazole prophylaxis starting in March 2008. A comparison was made between the two different periods (i.e., the pre-prophylaxis period, March 2003 through February 2008; and the prophylaxis period, March 2008 through February 2013).

The standard regimen for fluconazole prophylaxis was 3 mg/kg (Diflucan™, Pfizer Inc., Seoul, Korea or Oneflu™, JW Pharmaceutical, Seoul, Korea) administered once a day intravenously if a catheter was present, or through an orogastric tube, starting on the 3rd postnatal day [6], twice a week for 4 weeks. For cases of a presumed or proven IFI, fluconazole was suspended, and systemic antifungal therapy with non-liposomal or liposomal amphotericin B was administered empirically in both periods. For proven IFI, in uncomplicated cases, treatment was continued for 14 days after the last positive blood culture.

During the 10-year study period, there were no changes in infection control practices within both two NICUs. All NICU patients were screened regularly for occurrence of invasive infections, so that infants with antibiotics-resistant organism colonization could be placed in an isolation room with contact isolation procedures. Central venous catheters were placed and maintained sterilely according to the strict guidelines, and removals of their central venous catheters were discussed daily. All of the parenteral nutrition was prepared in pharmacy with aseptic technique and their qualities had been checked regularly on the base of the national administration regulations for level III centers.

### Identification of fungal colonization and infection

Surface cultures were routinely obtained from the following five body sites: the axilla, external ear canal, nasopharynx, throat (or tracheal aspirate if intubated) and anus. All of the procedures were performed with sterile transport swabs (COPAN Italia SpA, Brescia, Italy) or disposable respiratory specimen traps (HYUP SUNG Medical, Yangju, Kyunggi, Korea) for tracheal aspirates. The specimens were transported to microbiological laboratories within 30 min. Baseline surface cultures were obtained within 48 hours of birth from ELBW infants. Follow-up cultures were taken weekly for one month, and additional cultures were obtained weekly for infants with a central venous catheter. Fungal colonization was defined by a positive surveillance culture at any site, at any time during hospitalization. A positive culture from urine collected through urine bags (if <10^5^ CFUs/mL) was also considered indicative of fungal colonization.

For every episode of suspected sepsis, two or more blood samples from different sites were obtained, in addition to urine samples through either urine bags or an in/out catheter. The blood specimens were processed by clinical microbiology laboratories using a BacT/Alert 3D (Organon Teknika, Durham, NC, USA) system. For fungal isolation, Sabouraud-dextrose agar (SDA) and SDA-chloramphenicol plates were used. After isolation of grown fungi, yeasts were processed using a VITEK 2 system for both the identification and determination of antifungal susceptibility. If the result of susceptibility test using the VITEK 2 system was “resistant” or “intermediate”, an E-test was performed using Clinical Laboratory Standards Institute breakpoints. The interpretative breakpoints of fluconazole resistance were defined as ≥ 64 μg/mL, as recommended by National Committee for Clinical Laboratory Standards [16]. Molds were identified through morphological analysis after lacto-phenol cotton blue staining.

### Study outcomes

The primary outcome for the efficacy of fluconazole prophylaxis was the incidence of IFIs, which was defined by at least one positive culture from blood, urine (>10^5^ CFUs/mL in urine bags or 10^4^ CFUs/mL in an in/out catheter), ascites, pleural fluid or cerebrospinal fluid, accompanied by at least two clinical signs of systemic inflammatory responses (apnea, bradycardia, temperature ≥38.0 or ≤36.0 °C, blood sugar level ≥160 or ≤50 mg/dL). We also evaluated death from IFIs, the fungal colonization rate and respective species, the progression of fungal colonization to IFI, the length of NICU stay and mortality.

The primary safety outcome was the rates of IFIs involving fluconazole-resistant strains. We also measured the colonization rate of fluconazole-resistant fungi and the incidence of acute adverse events, including liver dysfunction (AST or ALT >250 IU/L) [17], renal dysfunction (creatinine >1.5 mg/dL), and cholestasis (direct bilirubin >2 mg/dL), which were assessed by weekly serum samples during the first 6 weeks. The incidence of skin rash, a known adverse reaction of fluconazole, was also determined. We collected data on rickets of prematurity based on a wrist X-ray at 4 weeks of age. Also included in analysis were late developmental morbidities (cerebral palsy, blindness, deafness and catch-up growth failure), which were assessed at 18 to 22 months of corrected age. Cerebral palsy was defined as a non-progressive disorder characterized by abnormal tone in at least one extremity and abnormal control of movement and posture. Blindness was defined as no functional vision in either eye. Deafness was defined as an inability to understand commands despite amplification, hearing aids, or cochlear implants in both ears. Failure of catch-up growth was defined as a weight of below the 10th percentile of the standardized growth curve.

### Statistical analysis

For 264 infants in the groups receiving fluconazole and 159 infant in the group not receiving fluconazole, we estimated that we would have at least 90 % power to detect differences in IFI rates based on previous reports [18, 19]. Statistical analysis was performed with SAS version 9.1.3 (SAS Institute, Cary, NC, USA) and SPSS version 21.0 (SPSS, Chicago, IL, USA). Categorical variables were analyzed using χ^2^ and Fisher’s exact test, as appropriate. Continuous variables were analyzed using the independent t-test and Wilcoxon rank sum test, as appropriate. Multivariate logistic regression was used to assess the significance of the variables. *P* <0.05 was considered statistically significant.

## Results

**Fig. 1:**
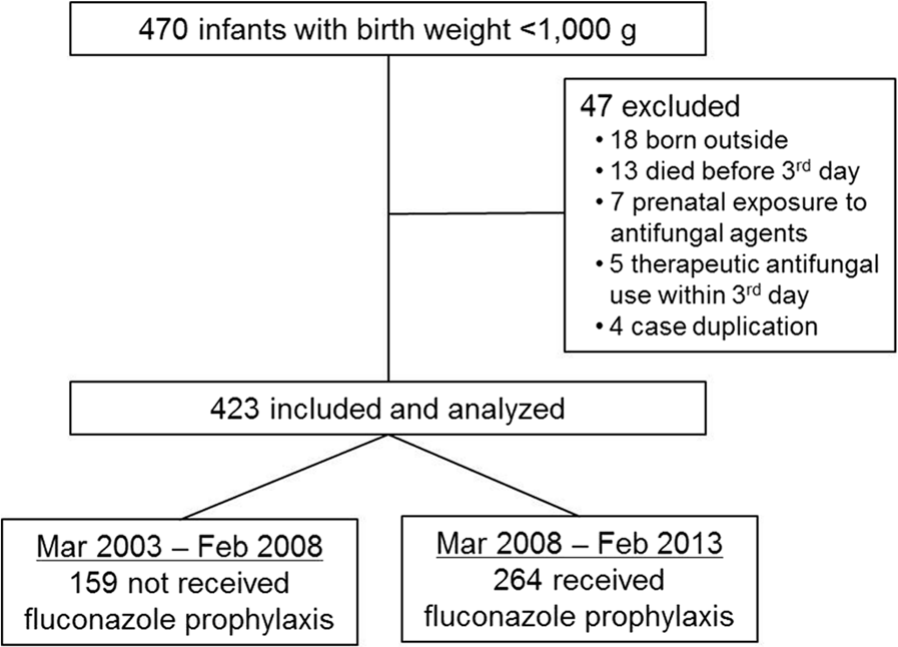
Flowchart of included extremely low birth weight infants during the study period

Of 470 ELBW infants during the study period, 47 were excluded from analysis. The fluconazole group consisted of 264 infants born during the period of prophylaxis use, and the pre-prophylaxis control group consisted of 159 infants who did not receive fluconazole prophylaxis (Fig. 1).

The demographic and clinical characteristics of both groups are shown in Table 1. There were no significant differences in the baseline characteristics between the two groups, except for antenatal steroid use. More infants were exposed to antenatal steroids in the fluconazole group (87 % vs. 72 %, *P* <0.001). Infants in the fluconazole group had fewer risk factors for fungal infection in their clinical courses, as defined by many previous reports; less frequently used H2 blockers (39 % vs. 69 %, *P* <0.001), 3rd generation cephalosporin (5 % vs. 26 %, *P* <0.001) and vancomycin (49 % vs. 60 %, *P* = 0.03) compared with those in the control group. The days to reach full enteral feeding (mean ± SD, 26 ± 16 vs. 31 ± 17 days, *P* = 0.001) and antibiotic duration (mean ± SD, 19 ± 20 vs. 28 ± 26 days, *P* <0.001) were also shorter in the fluconazole group compared with the control group, and the number of infants who received oxygen therapy for over 48 hours after birth was significantly lower in the fluconazole group (87 % vs. 94 %, *P* = 0.02).

**Table 1:** Demographic and clinical characteristics

	Pre-prophylaxis (*n* = 159)	Fluconazole prophylaxis (*n* = 264)	*P* value
Baseline characteristics			
Gestational age, mean ± SD, week	27^+1^ ± 2^+3^	27^+0^ ± 2^+1^	0.99
Birth weight, mean ± SD, g	761 ± 153	775 ± 154	0.32
Multiples	72 (45)	119 (45)	0.97
Vaginal delivery	42 (26)	80 (30)	0.39
Small for gestational age (<10 percentile)	69 (43)	112 (42)	0.85
Antenatal steroid use	108 (72)	229 (87)	<0.001
Antenatal antibiotic use	65 (41)	126 (48)	0.19
Maternal chorioamnionitis	46 (31)	97 (37)	0.16
Maternal preeclampsia	38 (24)	65 (25)	0.91
Maternal diabetes	7 (5)	7 (3)	0.32
Clinical courses			
Bronchopulmonary dysplasia	89 (60)	152 (63)	0.56
Necrotizing enterocolitis (surgical)	16 (10)	23 (9)	0.63
Intraventricular hemorrhage (≥ grade 2)	28 (18)	58 (22)	0.32
Retinopathy of prematurity	70 (49)	134 (56)	0.23
Invasive bacterial infection	47 (30)	82 (32)	0.67
Full feeding reach (≥100 mL/kg/d), mean ± SD, day	31 ± 17	26 ± 16	0.001
H2 blocker use	109 (69)	104 (39)	<0.001
Oxygen therapy > 48 hours	150 (94)	230 (87)	0.02
Intubation > 48 hours	127 (80)	198 (75)	0.28
Umbilical lines at postnatal 3rd day	22 (14)	35 (13)	0.85
Antibiotic duration, mean ± SD, day	28 ± 26	19 ± 20	<0.001
3rd generation cephalosporin use	42 (26)	12 (5)	<0.001
Vancomycin use	95 (60)	128 (49)	0.03

Values are reported as n (%) unless otherwise indicated

In a total of 19 ELBW neonates (4.7 %), 21 episodes of IFIs occurred during the 10-year study period. The distribution of fungal spp. causing infection, their sensitivities to fluconazole, and details of the IFIs are reported in Table 2. The most frequently identified species was *C. parapsilosis* (3 cases in the control group [42.9 %] and 9 in the fluconazole group [75.0 %], *P* = 0.33). Fluconazole-resistant *C. parapsilosis* was found in 5 cases (41.7 %) in the fluconazole group, and in none in the control group (*P* = 0.11). Although 28.6 % (2/7) of the IFIs were caused by *C. albicans* in the control group, there was only one patient with an IFI (8.3 %) caused by *C. albicans* in the fluconazole group (*P* = 0.52). Two infants infected with fluconazole-resistant *C. glabrata* eventually died as the direct result of the IFI, no matter which group they were involved.

**Table 2:** Details of invasive fungal infection events

Patient	Organism	Fluconazole-resistance	Onset day	Site of Infection	Mortality	Cause of Death
During the pre-prophylaxis period						
1	*Candida albicans*	Sensitive	8, 39	Blood	Died	Invasive fungal infection
2	*Candida parapsilosis*	Sensitive	18	Blood	Alive	
3	*Candida parapsilosis*	Sensitive	7	Blood	Alive	
4	*Candida parapsilosis*	Sensitive	13	Blood	Alive	
5	*Candida glabrata*	Resistant	13	Blood	Died	Invasive fungal infection
6	*Candida glabrata*	Sensitive	11	Blood	Alive	
7	*Candida albicans*	Sensitive	8	Blood, urine	Died	Invasive fungal infection
During the fluconazole prophylaxis period						
8	*Candida parapsilosis*	Sensitive	32	Blood, urine	Alive	
9	*Candida glabrata*	Resistant	19	Blood	Died	Invasive fungal infection
10	*Candida parapsilosis*	Resistant	24	Blood, ascites	Died	Cardiac failure, NEC^a^
11	*Candida parapsilosis*	Resistant	28	Blood	Died	Unknown
12	*Candida glabrata*	Sensitive	8	Blood	Alive	
13	*Candida parapsilosis*	Sensitive	21	Blood	Alive	
14	*Candida parapsilosis*	Sensitive	6	Blood	Alive	
15	*Candida albicans*	Sensitive	21	Blood, urine	Alive	
16	*Candida parapsilosis*	Resistant	43, 71	Blood	Alive	
17	*Candida parapsilosis*	Resistant	11	Blood	Alive	
18	*Candida parapsilosis*	Resistant	17	Blood	Alive	
19	*Candida parapsilosis*	Sensitive	12	Blood	Alive	

^a^ Necrotizing enterocolitis

### Efficacy outcomes

The primary efficacy outcome, IFI incidence was not significantly different between the two groups (5.0 % vs. 4.4 %, *P* = 0.80). This finding was consistent when applied to infants with birth weight <750 g (9.1 % vs. 8.8 %, *P* = 0.95). This result did not change when IFIs were evaluated as the number of events per 1,000 patient-days (Table 3).

**Table 3:** Efficacy outcomes of fluconazole prophylaxis

	Pre-prophylaxis	Fluconazole prophylaxis	*P* value
Primary outcomes			
Invasive fungal infection	7/159 (4.4)	12/242 (5.0)	0.80
Invasive fungal infection in birth weight <750 g	6/68 (8.8)	9/99 (9.1)	0.95
Invasive fungal infection, event per 1,000 patient days	0.56	0.56	0.99
Secondary outcomes			
Death	26/159 (16.4)	31/264 (11.7)	0.18
Death from invasive fungal infection	3/26 (11.5)	1/31 (3.2)	0.32
Invasive fungal infection related death	2/7 (28.6)	1/12 (8.3)	0.52
Baseline fungal colonization	4/149 (2.7)	3/224 (1.3)	0.71
Fungal colonization	88/149 (59.1)	76/224 (33.9)	<0.001
Single:Multiple(≥2 sites)	21:67	17:59	0.44
Axilla:Ear:Nasopharynx:Throat/trachea:Anus:Urine	46:25:2:8:29:14	33:19:4:10:19:15	-
Fungal colonization to invasive infection	4/88 (4.6)	6/76 (7.9)	0.52
Length of hospitalization, mean ± SD, day	90 ± 51	88 ± 52	0.74

Values are reported as n/total (%) unless otherwise indicated

Mortality and the rates of death from an IFI did not differ between the two groups (11.7 % vs. 16.4 %, *P* = 0.18 and 3.2 % vs. 11.5 %, *P* = 0.32, respectively). The fungal colonization rate was significantly lower in the fluconazole group (33.9 % vs. 59.1 %, *P* <0.001). However, the conversion ratio of fungal colonization to invasive infection did not differ between the two groups (7.9 % vs. 4.6 %, *P* = 0.52). The hospitalization duration was similar between the two groups (mean ± SD, 88 ± 52 vs. 90 ± 51 days, *P* = 0.74).

Post hoc analysis to evaluate the impact of fluconazole use for IFI prophylaxis demonstrated that it did not decrease IFI risk (adjusted *OR* = 1.92, 95 % *CI* = 0.30-12.23, *P* = 0.49). Multivariate logistic regression analysis revealed that the risk factors for IFI were low gestational age (adjusted *OR* = 0.87, 95 % *CI* = 0.80-0.95, *P* = 0.002), a longer duration to reach full enteral feeding (adjusted *OR* = 1.06, 95 % *CI* = 1.02-1.09, *P* = 0.001), and necrotizing enterocolitis requiring surgical intervention (adjusted *OR* = 7.34, 95 % *CI* = 2.00-26.95, *P* = 0.003) (Table 4).

**Table 4:** Analysis on the risk for invasive fungal infection

	Unadjusted OR (95 % CI)	Adjusted OR^a^ (95 % CI)	*P* value^b^
Fluconazole prophylaxis	1.13 (0.43–2.94)	1.92 (0.30–12.23)	0.49
Gestational age	0.90 (0.86–0.95)	0.87 (0.80–0.95)	0.002
Birth weight	1.0 (0.99–1.0)	1.0 (0.99–1.01)	0.76
Antepartum antibiotic use	7.13 (2.04–24.9)	10.85 (0.94–125.50)	0.06
Umbilical line at postnatal 3rd day	4.44 (1.65–11.95)	0.68 (0.10–4.86)	0.70
H2 blocker use	3.83 (1.25–11.75)	0.76 (0.09–6.09)	0.79
Full feeding reach (≥100 mL/kg/d)	1.05 (1.03–1.08)	1.06 (1.02–1.09)	0.001
Necrotizing enterocolitis (surgical)	8.34 (3.12–22.31)	7.34 (2.00–26.95)	0.003
Invasive bacterial infection	4.89 (1.81–13.18)	1.55 (0.29–8.37)	0.61
Antibiotic duration	1.03 (1.02–1.05)	1.02 (1.00–1.05)	0.09
Vancomycin use	16.55 (2.19–125.23)	3.63 (0.30–43.33)	0.31

^a^ Multivariate logistic regression analysis was done with stepwise method

^b^ for adjusted OR

### Safety outcomes

The proportion of IFIs caused by fluconazole-resistant strains was higher in the fluconazole group compared with the control group, but this difference did not reach statistical significance (50.0 % vs. 14.3 %, *P* = 0.17). IFIs involving natively fluconazole-resistant strains (*C. krusei* and *C. glabrata*) were not more frequent during fluconzole prophylxis. IFIs involving fluconazole-resistant *C. parapsilosis* were much increased during the fluconazole prophylaxis period, but also did not reach statistical significance (41.7 % vs. 0 %, *P* = 0.11). Colonization by fluconazole-resistant fungi and natively fluconazole-resistant strains was similar between the two groups. There were no between-group differences in the number of infants with liver dysfunction, renal dysfunction, cholestasis, rickets of prematurity, or rash. Late morbidities evaluated at 18 to 22 months corrected age, including cerebral palsy, blindness, deafness and catch up growth failure, were not significantly different between the two groups (Table 5).

**Table 5:** Safety outcomes of fluconazole prophylaxis

	Pre-prophylaxis	Fluconazole prophylaxis	*P* value
Invasive fungal infection by fluconazole-resistant strains	1/7 (14.3)	6/12 (50.0)	0.17
Invasive fungal infection by natively fluconazole-resistant strains^a^	2/7 (28.6)	2/12 (16.7)	0.60
Invasive fungal infection by fluconazole-resistant *C. parapsilosis*	0/7 (0)	5/12 (41.7)	0.11
Fungal colonization by fluconazole-resistant strains	4/25 (16.0)	9/55 (16.4)	>0.99
Fungal colonization by natively fluconazole-resistant strains^a^	10/88 (11.4)	9/76 (11.8)	0.92
Acute adverse events			
Liver dysfunction (AST or ALT > 250 U/L)^b^	8/156 (5.1)	11/262 (4.2)	0.66
Renal dysfunction (creatinine > 1.5 mg/dL)^b^	34/155 (21.9)	46/262 (17.6)	0.27
Cholestasis (direct bilirubin > 2 mg/dL)^b^	10/74 (13.5)	24/238 (10.1)	0.41
Skin rash^b^	10/150 (6.7)	16/262 (6.1)	0.84
Rickets of prematurity^c^	24/100 (24.0)	70/177 (39.4)	0.63
Late morbidities at 18 to 22 months corrected age			
Cerebral palsy	8/126 (6.4)	28/222 (12.6)	0.11
Blindness, at least one eye	0/131 (0)	0/230 (0)	-
Deafness, at least one ear	0/132 (0)	2/227 (0.9)	0.54
Failure to catch up growth (<10 percentile)	52/108 (48.2)	87/192 (45.3)	0.64

Values are reported as n/total (%) unless otherwise indicated

^a^ *C. krusei and C. glabrata*

^b^ Episodes occurred during 6 weeks after birth were counted

^c^ Documented by the wrist X-ray at 4 weeks of age

## Discussion

In this retrospective cohort analysis conducted over a 10-year period, although it lowered the fungal colonization rate significantly, fluconazole prophylaxis did not reduce the incidence of IFIs in ELBW infants. In addition, although the increase did not reach statistical significance, fluconazole prophylaxis tended to increase the incidence of invasive infections involving fluconazole-resistant *C. parapsilosis* (41.7 % vs. 0 %, *P* = 0.11). This discrepant result might be related to other infection control areas or presence of other source of fungal infection during fluconazole prophylaxis period. However, there were no changes in infection control practices during whole study periods and the incidence of invasive bacterial infection was similar between two periods. In addition, the fact that fluconazole-received infants had less exposure to well-known risk factors for IFIs (e.g., the prolonged use of broad spectrum antibiotic, H2 blocker, and delayed enteral feeding completion) raise the question regarding the efficacy of fluconazole prophylaxis.

In agreement with other investigations, our study showed that advanced necrotizing enterocolitis and a longer duration to reach to full enteral feeding increased the odds of developing an IFI. However, fluconazole prophylaxis did not decrease the risk of IFI development (adjusted *OR* = 1.92, 95 % *CI* = 0.3-12.23) in ELBW infants. Notably, the IFI incidences markedly varied between the NICUs (ranging from <2 to 30 %) [3–10, 20, 21], and the potential benefits of fluconazole prophylaxis may be less in centers with a low incidence of IFIs. The pre-prophylaxis period exhibited a 4.4 % (unit 1, 4.7 %; unit 2, 3.3 %) incidence of proven invasive candidiasis in this study. Fluconazole has been shown to be very effective in preventing candidiasis in previous studies with high incidences of IFIs (13 % [6] and 20 % [5] in the fluconazole-untreated groups). However, the fluconazole use for antifungal prophylaxis in NICUs with a low incidence of IFIs is controversial. The latest guidelines of the European Society of Clinical Microbiology and Infectious Diseases (2012) recommend fluconazole prophylaxis of 3-6 mg/kg/dose twice weekly intravenously or orally for neonates <1,000 g only for NICUs with relatively high frequency of IFIs [22]. For NICUs with a lower incidence (<2 %) of IFIs, this guideline recommends that the decision should be made on a case-by-case basis and embedded in a risk stratification strategy (e.g., additional risk factors for IFIs, such as central venous catheterization and the receipt of third-generation cephalosporins or carbapenems) [22].

There is concern regarding the emergence of a fungal ecological shift toward *Candida* spp. (*C. glabrata* or *C. krusei*) with intrinsic resistance to fluconazole [13, 23]. In this study, the routine administration of fluconazole prophylaxis for 4 weeks to all ELBW infants for a period of 5 years did not lead to selection for the colonization with *C. glabrata or C. krusei*, which are natively resistant to fluconazole (Table 5). However, from the non-prophylaxis era to the fluconazole prophylaxis period, we detected a shift in *Candida* species causing invasive infection from a majority of *C. albicans* to entirely non-albicans species. There was a decrease in the rate of invasive infection by *C. albicans* (28.6 % to 8.3 %), with a corresponding increase in *C. parapsilosis* infections (42.9 % to 75.0 %) and its fluconazole resistance (0 % to 55.6 %) after routine fluconazole exposure over a 5-year period (Table 2). This change in the distribution of *Candida* species might be concomitant with a reduction in *C. albicans*-attributable infections, given that this species is exquisitely susceptible to fluconazole. However, one animal study has reported an increase in the occurrence of fluconazole-resistant *C. parapsilosis* infections after a 4-year period of antifungal prophylaxis in a premature animal NICU [24]. Similarly, Sarvikivi et al. reported that the prolonged (>10 years) use of fluconazole for prophylaxis in a NICU resulted in the emergence of resistant *C. parapsilosis* [25]. In addition, a negative correlation has been found between fluconazole consumption and the rate of *C. parapsilosis* bloodstream infections [25]. Moreover, in an Indian NICU at which fluconazole prophylaxis had been used routinely for the preceding 6 years, the most common fungal isolates causing invasive infections were non-albicans *Candida* species with relatively reduced azole susceptibility [26]. This pattern of resistance was also observed in critically ill adults who had received antifungal prophylaxis [27].

However, many studies that evaluate the effect of fluconazole prophylaxis in preterm infants have not observed any statistically significant difference on fungal resistance patterns [13, 19, 28–30]. Although it is guessed several known mechanisms, which has been described as manners of acquired resistance for the azoles in *Candida* spp., might act in concert leading to stepwise increases in minimum inhibitory concentrations (MICs) and broadening of the azole resistance spectrum during long term exposure to fluconazole [31–33], there is conflict on how it may impact to local epidemiology trends in *Candida* spp. with a cross-patient delivery of antifungal resistance in NICUs. Additionally, as *C. parapsilosis* is associated with parenteral nutirion preparation and possible horizontal transmission via NICU staffs, infection preventive measures of each unit might be more contributable to the pattern changes of local epidemiology in *Candida* spp. Ten-year data from the National Nosocomial Infections Surveillance System of the United States has demonstrated decreases in the incidences of candidemia and invasive infections due to both *C. albicans* and *C. parapsilosis* in ELBW infants, and no changes in the incidences of infections involving other *Candida* spp [34]. There is a need for prospective studies to measure the long-term effect of fluconazole prophylaxis on antifungal resistance patterns. Additionally, on-going local and national efforts to detect the emergence of resistant organisms are needed [7].

Several limitations of this study should be considered. First, this was an uncontrolled observational study conducted in two NICUs. Confounding factors cannot be excluded, but the roles of any possible unidentified confounding variables are thought to be minimal and infection control policies were consistently applied for all infants during the study period. Second, our study design did not include routine MIC testing or the subtyping of DNA from obtained isolates. Third, potentially starting fluconazole prophylaxis earlier, on the day of life 1 or 2 would work better than 3 day in this study. There is a report that demonstrates the superior efficacy when starting antifungal prophylaxis within 48 hours after birth, prior to colonization [35]. At last, it is difficult to evaluate the actual culturing activity in NICUs over such a long time period. Invasive candidiasis is detected at a low sensitivity in blood cultures [36]. The small blood volumes used in the culture studies may have exacerbated this low diagnostic sensitivity and caused the selective under-diagnosis of IFIs in premature infants. For ascertaining the hidden effects of IFIs, we evaluated all-cause mortality, which is not affected by this bias, and found no statistically significant effect of fluconazole prophylaxis on mortality rates.

## Conclusions

Our results discourage the routine use of fluconazole prophylaxis for ELBW infants in NICUs with a low IFI incidence. However this study is one of many studies that evaluate the effect of fluconazole prophylaxis in preterm infants, in which mostly have not demonstrated resistance. Even if preventing invasive *Candida* infections that are associated with high mortality and neurodevelopemental impairment needs to remain a focus in ever NICU, its long-term effects should be carefully evaluated. Having knowledge of local epidemiologic trends in *Candida* spp. and monitoring of fungal isolates for drug resistance would be essential and further prospective studies are needed to examine fluconazole resistance patterns in premature infants.

## Availability of data and materials

The datasets supporting the conclusions of this article are included as additional files (see Additional file 1 and Additional file 2).

## Additional files

Additional file 1: Datasets. Excel data were retrieved from the data repository. (XLS 1976 kb) Additional file 2: Data dictionary. The coding IDs, names, sequences and values of each data are described. (XLSX 2247 kb)

## Abbreviations

ELBW: extremely low birth weight infant
IFI: invasive fungal infection
MIC: minimum inhibitory concentration
NICU: neonatal intensive care unit

## References

[1] Benjamin DK, Stoll BJ, Fanaroff AA, McDonald SA, Oh W, Higgins RD (2006). Neonatal candidiasis among extremely low birth weight infants: risk factors, mortality rates, and neurodevelopmental outcomes at 18 to 22 months. Pediatrics.

[2] Kaufman DA, Manzoni P (2010). Strategies to prevent invasive candidal infection in extremely preterm infants. Clin Perinatol.

[3] Benjamin DK, Stoll BJ, Gantz MG, Walsh MC, Sanchez PJ, Das A (2010). Neonatal candidiasis: epidemiology, risk factors, and clinical judgment. Pediatrics.

[4] Aydemir C, Oguz SS, Dizdar EA, Akar M, Sarikabadayi YU, Saygan S (2011). Randomised controlled trial of prophylactic fluconazole versus nystatin for the prevention of fungal colonisation and invasive fungal infection in very low birth weight infants. Arch Dis Child Fetal Neonatal Ed.

[5] Kaufman DA, Boyle R, Hazen KC, Patrie JT, Robinson M, Donowitz LG (2001). Fluconazole prophylaxis against fungal colonization and infection in preterm infants. N Eng J Med.

[6] Manzoni P, Stolfi I, Pugni L, Decembrino L, Magnani C, Vetrano G (2007). A multicenter, randomized trial of prophylactic fluconazole in preterm neonates. N Eng J Med.

[7] Austin N, McGuire W (2013). Prophylactic systemic antifungal agents to prevent mortality and morbidity in very low birth weight infants. Cochrane Database Syst Rev.

[8] Kossoff EH, Buescher ES, Karlowicz MG (1998). Candidemia in a neonatal intensive care unit: trends during fifteen years and clinical features of 111 cases. Pediatr Infect Dis J.

[9] Clerihew L, Lamagni TL, Brocklehurst P, McGuire W (2006). Invasive fungal infection in very low birth weight infants: national prospective surveillance study. Arch Dis Child Fetal Neonatal Ed.

[10] Vergnano S, Menson E, Kennea N, Embleton N, Russell AB, Watts T (2011). Neonatal infections in England: the NeonIN surveillance network. Arch Dis Child Fetal Neonatal Ed.

[11] Aliaga S, Clark RH, Laughon M, Walsh TJ, Hope WW, Benjamin DK (2014). Changes in the incidence of candidiasis in neonatal intensive care units. Pediatrics.

[12] Fanaroff AA (2006). Fluconazole for the prevention of fungal infection: get ready, get set, caution. Pediatrics.

[13] Manzoni P, Leonessa M, Galletto P, Latino MA, Arisio R, Maule M (2008). Routine use of fluconazole prophylaxis in a neonatal intensive care unit does not select natively fluconazole-resistant *Candida* subspecies. Pediatr Infect Dis J.

[14] Chicella MF, Woodruff ED, Desai MM (2012). A review of *Candida* prophylaxis in the neonatal intensive care population. J Pediatr Pharmacol Ther.

[15] Castagnola E, Jacqz-Aigrain E, Kaguelidou F, Maragliano R, Stronati M, Rizzollo S (2012). Fluconazole use and safety in the nursery. Early Hum Dev.

[16] National Committee for Clinical Laboratory Standards (2002). Reference method for broth dilution antifungal susceptibility testing of yeasts; Approved standard, M27-A.

[17] Aghai ZH, Mudduluru M, Nakhla TA, Amendolia B, Longo D, Kemble N (2006). Fluconazole prophylaxis in extremely low birth weight infants: association with cholestasis. J Perinatol.

[18] Rueda K, Moreno MT, Espinosa M, Saez-Llorens X (2010). Impact of routine fluconazole prophylaxis for premature infants in a developing country. Pediatr Infect Dis J.

[19] Manzoni P, Arisio R, Mostert M, Leonessa M, Farina D, Latino MA (2006). Prophylactic fluconazole is effective in preventing fungal colonization and fungal systemic infections in preterm neonates: a single-center, 6-year, retrospective cohort study. Pediatrics.

[20] Kim CS, Hong SA, Lee SL, Kim HS (2010). Effect of fluconazole prophylaxis to control *Candida* infection in high-risk preterm infants. Korean J Perinatol.

[21] Kim SY, Lee SJ, Kim MJ, Song ES, Choi YY (2007). Fluconazole prophylaxis in high-risk, very low birth weight infants. Korean J Pediatr.

[22] Hope WW, Castagnola E, Groll AH, Roilides E, Akova M, Arendrup MC (2012). ESCMID guideline for the diagnosis and management of *Candida* disease 2012: prevention and management of invasive infections in neonates and children caused by *Candida* spp. Clin Microbiol Infect.

[23] Hope W, Morton A, Eisen DP (2002). Increase in prevalence of nosocomial non-*Candida albicans* candidaemia and the association of *Candida krusei* with fluconazole use. J Hosp Infect.

[24] Yoder BA, Sutton DA, Winter V, Coalson JJ (2004). Resistant *Candida parapsilosis* associated with long term fluconazole prophylaxis in an animal model. Pediatr Infect Dis J.

[25] Sarvikivi E, Lyytikäinen O, Soll DR, Pujol C, Pfaller MA, Richardson M (2005). Emergence of fluconazole resistance in a *Candida parapsilosis* strain that caused infections in a neonatal intensive care unit. J Clin Microbiol.

[26] Parikh TB, Nanavati RN, Patankar CV, Rao S, Bisure K, Udani RH (2007). Fluconazole prophylaxis against fungal colonization and invasive fungal infection in very low birth weight infants. Indian Pediatr.

[27] Safran DB, Dawson E (1997). The effect of empiric and prophylactic treatment with fluconazole on yeast isolates in a surgical trauma intensive care unit. Arch Surg.

[28] Kaufman DA, Boyle R, Hazen KC, Patrie JT, Robinson M, Grossman LB (2005). Twice weekly fluconazole prophylaxis for prevention of invasive *Candida* infection in high-risk infants of <1000 grams birth weight. J Pediatr.

[29] Blot S, Janssens R, Claeys G, Hoste E, Buyle F, De Waele JJ (2006). Effect of fluconazole consumption on long-term trends in candidal ecology. J Antimicrob Chemother.

[30] Healy CM, Campbell JR, Zaccaria E, Baker CJ (2008). Fluconazole prophylaxis in extremely low birth weight neonates reduces invasive candidiasis mortality rates without emergence of fluconazole-resistant *Candida* species. Pediatrics.

[31] Sanglard D, Coste A, Ferrari S (2009). Antifungal drug resistance mechanisms in fungal pathogens from the perspective of transcriptional gene regulation. FEMS Yeast Res.

[32] Arendrup MC (2013). *Candida* and Candidaemia susceptibility and epidemiology. Dan Med J.

[33] Li X, Brown N, Chau AS, Lopez-Ribot JL, Ruesga MT, Quindos G (2004). Changes in susceptibility to posaconazole in clinical isolates of *Candida albicans*. J Antimicrob Chemother.

[34] Fridkin SK, Kaufman D, Edwards JR, Shetty S, Horan T (2006). Changing incidence of *Candida* bloodstream infections among NICU patients in the United States: 1995-2004. Pediatrics.

[35] Ozturk MA, Gunes T, Koklu E, Cetin N, Koc N (2006). Oral nystatin prophylaxis to prevent invasive candidiasis in neonatal intensive care unit. Mycoses.

[36] Berenguer J, Buck M, Witebsky F, Stock F, Pizzo PA, Walsh TJ (1993). Lysis-centrifugation blood cultures in the detection of tissue-proven invasive candidiasis. Diagn Microbiol Infect Dis.

